# Case Study - Candida auris in Skilled Nursing Facility

**DOI:** 10.1093/geroni/igab046.2340

**Published:** 2021-12-17

**Authors:** Nancy Vo, Sayaka Tokumitsu, Armen Melik-Abramians, S Chen John

**Affiliations:** 1 Kaiser Permanente Los Angeles Medical Center, California, United States; 2 Kaiser Permanente Med Group/Southern California, Los Angeles, California, United States

## Abstract

Candida Auris (C. auris), is a multidrug-resistant organism, first described in Japan 2009, and now a serious, emerging global health threat1. C. auris pathogen can potentiate morbidity and mortality, i.e. lifelong contact precaution isolation, intravenous antifungal treatment, hospitalization and mortality rate of 30-60%1. Los Angeles County (LAC) developed 15 new cases in May 2020, and 73 cases in July 2020, amidst COVID-19 pandemic2. A 88 year old Black female had a positive skin test for C. auris by LAC Department of Public Health (DPH) during skilled nursing facility (SNF) admission for hip fracture in September 2020. Patient’s risk factors for C. auris included: age, kidney transplantation (1998) immunosuppression on tacrolimus, fungal infection on fluconazole, drug-drug interaction between tacrolimus-fluconazole including nephrotoxicity and neurotoxicity, malnutrition, bedbound, Stage 4 sacrococcyx pressure ulcer, osteomyelitis on broad-spectrum antibiotics, chronic indwelling catheter, and healthcare setting. Our multimorbid and frail patient remained asymptomatic with C. auris under an interdisciplinary team approach, including geriatricians, infectious disease, pharmacists, SNF team and local DPH. Our patient’s psychosocial isolation and family distress with local DPH guidelines for COVID-19 SNF visitation restrictions were compounded by multifaceted coordination of patient-centered care between SNF team and specialists via telehealth. Further research in the prevention, detection, and management of C. auris is warranted to protect our vulnerable SNF residents. 1. Centers for Disease Control and Prevention. (2020). Tracking Candida auris. https://www.cdc.gov/fungal/candida-auris/tracking-c-auris.html 2. Los Angeles County Health Alert Network. (2020). CDPH Health Advisory: Resurgence of Candida auris in Healthcare Facilities in the Setting of COVID-19. http://publichealth.lacounty.gov/eprp/lahan/alerts/CAHANCauris082020.pdf

